# Come back when you’re infected: pharmacy access to sterile syringes in an Arizona Secret Shopper Study, 2023

**DOI:** 10.1186/s12954-024-00943-w

**Published:** 2024-02-22

**Authors:** Danielle M. Russell, Beth E. Meyerson, Arlene N. Mahoney, Irene Garnett, Chris Ferrell, Kylee Newgass, Jon D. Agley, Richard A. Crosby, Keith G. Bentele, Nina Vadiei, David Frank, Linnea B. Linde-Krieger

**Affiliations:** 1 Arizona State University, Tempe, AZ USA; 2 Harm Reduction Research Lab, University of Arizona College of Medicine-Tucson, Tucson, AZ USA; 3 Family and Community Medicine, College of Medicine, University of Arizona, Tucson, AZ USA; 4 Southwest Recovery Alliance, Phoenix, AZ USA; 5 Drug Policy Research and Advocacy Board (DPRAB), University of Arizona, Tucson, AZ USA; 6 CAN Community Health, Phoenix, AZ USA; 7 Indiana University School of Public Health-Bloomington, Bloomington, IN USA; 8 University of Kentucky College of Public Health, Lexington, KY USA; 9 Southwest Institute for Research On Women, University of Arizona, Tucson, AZ USA; 10 College of Pharmacy, University of Arizona, Tucson, AZ USA; 11 School of Global Public Health, New York University, New York, USA; 12 Center for Comprehensive Pain and Addiction, University of Arizona Health Sciences, Tucson, AZ USA

**Keywords:** Syringes, Hepatitis C, Community pharmacies, Evidence-based pharmacy practice, Public health, HIV infections, Delivery of health care

## Abstract

**Background:**

Pharmacies are critical healthcare partners in community efforts to eliminate bloodborne illnesses. Pharmacy sale of sterile syringes is central to this effort.

**Methods:**

A mixed methods “secret shopper” syringe purchase study was conducted in the fall of 2022 with 38 community pharmacies in Maricopa and Pima Counties, Arizona. Pharmacies were geomapped to within 2 miles of areas identified as having a potentially high volume of illicit drug commerce. Daytime venue sampling was used whereby separate investigators with lived/living drug use experience attempted to purchase syringes without a prescription. Investigator response when prompted for purchase rationale was “to protect myself from HIV and hepatitis C.” A 24-item instrument measured sales outcome, pharmacy staff interaction (hostile/neutral/friendly), and the buyer’s subjective experience.

**Results:**

Only 24.6% (*n* = 28) of 114 purchase attempts across the 38 pharmacies resulted in syringe sale. Less than one quarter (21.1%) of pharmacies always sold, while 44.7% never sold. Independent and food store pharmacies tended not to sell syringes. There emerged distinct pharmacy staff interactions characterized by body language, customer query, normalization or othering response, response to purchase request and closure. Pharmacy discretion and pharmacy policy not to sell syringes without a prescription limited sterile syringe access. Investigators reported frequent and adverse emotional impact due to pharmacy staff negative and stigmatizing interactions.

**Conclusions:**

Pharmacies miss opportunities to advance efforts to eliminate bloodborne infections by stringent no-sale policy and discretion about syringe sale. State regulatory policy facilitating pharmacy syringe sales, limiting pharmacist discretion for syringe sales, and targeting pharmacy-staff level education may help advance the achievement of public health goals to eliminate bloodborne infections in Arizona.

**Supplementary Information:**

The online version contains supplementary material available at 10.1186/s12954-024-00943-w.

## Introduction

It has been estimated that about 10% of the US population has struggled to navigate their relationship with intoxicants at some point in their lifetime [1].These estimates indicate that a potentially sizable number of individuals in the USA may use illicit drugs. Stigma and marginalization can have potentially lethal impact on the health and well-being of people who use illicit drugs, including people who inject illicit drugs (PWID). Healthcare settings are primary venues in which PWID often report experiencing stigma, improper medical care, neglect, and abuse by healthcare providers [2–5]. Such experiences can result in healthcare-seeking delays, thereby exacerbating medical conditions [6, 7]. In addition to physical health implications, these experiences also cause unnecessary and preventable suffering and trauma, which the director of the US National Institute on Drug Abuse (NIDA) has called “a powerful social penalty [8].” PWID are at increased risk for bloodborne infections (HIV, HCV), skin and soft tissue infections (SSTI), compartment syndrome, and endocarditis as a result of not having adequate access to sterile injection equipment [9]. In 2021, 8% of all new US-based HIV infections were among PWID, up from 7% in 2020 [10]. Injection-related infections and diseases contribute to increased mortality among PWID and are fueled by lack of preventive health services and healthcare access. Syringe service programs (SSP) can help mitigate injection-related diseases and infections [11–13]. However, in the US, SSP are geographically maldistributed and limited due to sporadic and insufficient funding [14, 15]. As a result, because they are also in almost every US community, many PWID seek to purchase sterile syringes in community pharmacies [16]. The ability of community pharmacies to increase access to sterile syringes cannot be overstated. A study in Massachusetts and Rhode Island found that in a 1-year period, a single pharmacy chain sold approximately 10 times the number of sterile syringes distributed by all in those states combined [17].

According to the Network for Public Health Law, most states allow retail sale of syringes without prescription, and some states like Arizona (the state where this study was conducted) do not explicitly regulate retail syringe sales to adults [18]. Despite the policy environment, studies demonstrate that many pharmacists elect not to sell syringes without a prescription and this trend may be increasing. A 2004 study in Colorado, Kentucky, Missouri and Connecticut found that 65% of requests to purchase syringes in community pharmacies without a prescription were honored [19]. Over a decade later in 2016, an Indiana study found that 51% of community pharmacy managers reported having dispensed syringes without a prescription between 2014 and 2016 [20]. Two years later and following the largest US-based HIV outbreak among PWID in decades (occurring in Indiana), only 29% of community pharmacy managers reported personally having dispensed syringes in the previous 2 years (2016–2018) [21, 22]. An analysis of Indiana’s HIV outbreak indicated that had sterile syringes been available; more than half of the HIV infections would have been prevented [23]. Notably, this observed pharmacy practice trend is counter to the most recent (2016) accreditation standards for a Doctor of Pharmacy degree embracing patient-centered care and wellness practices [24].

These pharmacy patterns are also observed in Arizona. A 2018 study found that only 10% of interview participants had been sold syringes without a prescription in community pharmacies in the prior 2 years (2016–2018), with sales outcome variations at the same pharmacy and pharmacy chain [25]. Sales variation was also observed by pharmacy type in a survey among Arizona pharmacists conducted that same year, in which a majority (75%) of mass merchandizer pharmacies reported dispensing syringes without a prescription in the past 2 years, compared to 58% of chain pharmacies, 28% of food store pharmacies and 8.3% of independent pharmacies [26]. A comparable study published in 2015 found that only 21% of syringe purchase attempts were successful across 248 California community pharmacies in Fresno and Kern counties [27]. These findings suggest the possibility that pharmacy discretion, or pharmacist discretion specifically, may affect access to sterile syringes in Arizona and therefore health outcomes. The Arizona findings are concerning considering that Maricopa County, Arizona (home to the City of Phoenix), is among the top counties contributing more than 50% of new HIV infections in the USA [28, 29].

These health outcomes and risks have led the state of Arizona to establish policy to eliminate viral hepatitis [30]. Success will likely depend in part upon community pharmacies. This study sought to document current pharmacy practice using a secret shopper methodology to inform Arizona policy efforts to reduce bloodborne illnesses and eliminate viral hepatitis.

## Methods

A secret shopper syringe purchase study was conducted in two Arizona counties: Maricopa and Pima. A list of community pharmacies provided by Hayes Directories Inc (Mission Viejo, CA) was geographically cross referenced with areas identified by a local harm reduction organization where illicit drug commerce might be prevalent. Geospatial mapping was used to approximate the need for syringe purchase based on pharmacy proximity. Pharmacies within a 2-mile radius were selected for the study based on participant feedback from a 2019 Arizona syringe study finding that people would walk up to 2 miles to purchase sterile syringes from a pharmacy to protect their health [31]. There were 38 pharmacies identified by this method, 29 in Maricopa County and 9 in Pima County (home to Tucson). A daytime venue sampling strategy was used to assure that pharmacies would be visited on three separate occasions, at different times of day, during different weeks by three separate field investigators.

A 24-item instrument measured syringe sales outcome (yes/no) and the tone of the pharmacy staff interaction with the field investigator posing as a buyer using a scale of friendly/neutral/negative first used by Compton in 2004 [19]. The novel addition by the study reported here was the inclusion of field investigator subjective experience as people with lived/living drug use experience. This approach, adapting Meyerson’s 2014 secret shopper HIV testing study, characterized the impact of the experience on the buyer because such experiences have been shown to impact future healthcare or preventive care-seeking decisions [25, 30, 31].

Field investigators represented a range of age groups, gender, and racial identities. The criteria for investigator hiring included living in Pima or Maricopa Counties, being over 18 years or older, and having lived/living drug use experience. Investigators were trained in research ethics and human subjects protection with study oversight by the University of Arizona Institutional Review Board.

The protocol was as follows: investigators entered the assigned pharmacy and requested to purchase “*a bag of 10 syringes*.” If pharmacy staff inquired about their purpose, investigators replied, “*I need them to protect myself from HIV and Hepatitis C*.” Investigators were instructed to be regular pharmacy customers and not to reveal their identity as investigators studying the pharmacy. They were asked to visit the pharmacy as they are. There were no directions about what to wear or how to present themselves beyond asking to purchase syringes for their health protection. This direction recognized that people purchase pharmacy services in all manner of clothing or presentation, even as the 2019 Arizona pharmacy study participants judged themselves regarding how they might have physically appeared to the pharmacist and how that may have caused the syringe sale refusal [25]. Following interaction with pharmacy staff, field investigators documented the sales outcome and responded to the instrument items using a provided handheld audio recorder within 30 min of the pharmacy visit. Audio recordings were uploaded to a secure University of Arizona server within 24 h and immediately confirmed for transcription. Because negative interactions with pharmacy staff can be distressing, the study structure included online video ‘drop in’  opportunities and text communication led by author (DMR) to provide emotional support to field investigators as needed.

Sales outcomes were calculated by pharmacy and pharmacy type and coded for consistency across the three visits using the following categories: ‘sold at least once,’ ‘sold at least twice,’ ‘always sold,’ and ‘never sold.’ An additional category, ‘only sold box’ (of 100 syringes), was added based on investigator experiences. Quantitative data were managed and analyzed using SPSS version 28 (IBM Corp).

Qualitative data were managed by a codebook developed iteratively by three independent researchers (DMR, BEM, and ANM). An initial codebook contained *a priori *indicators from the instrument such as sales outcome and pharmacy staff tone. The second iteration containing additional codes and structures emerged following independent coding of data from a random sample of 15 pharmacy visits. A coding conference was held and minor discrepancies were identified and managed through discussion. A final codebook was established and used to code all field visits by DMR. The two other researchers (BEM, ANM) each coded a randomly selected 1/3 of the field visits. Emerging codes were then reviewed in conference and collapsed into major categories of “Buyer Experience” and “Staff Response.” Finally, relevant codes were then arrayed by category with major themes reported. Field investigators met to discuss, clarify, and prioritize findings with DMR, BEM, and ANM for reporting here. All qualitative data were managed and analyzed using QSR NVIVO 14 (QSR International Pty Ltd., 2020).

## Results

### Pharmacy syringe sales

There were 12 distinct pharmacy organizations across the four pharmacy types in the sample of 38 pharmacies. Food store pharmacies (*n* = 10; 26.3%) included Basha’s Pharmacy, Fry’s pharmacy (Kroger), and Safeway pharmacy. Mass merchandiser pharmacies (*n* = 5; 13.2%) included Walmart pharmacy and Target/CVS pharmacy. Retail chain pharmacies (*n* = 19; 50%) included CVS, Walgreen Drug Store, and Community/Walgreens Drug store. Target/CVS was categorized as a mass merchandizer as opposed to the stand-alone CVS retail chain pharmacy to reflect prior observed pharmacy practice environment differences between stand-alone CVS pharmacies and CVS pharmacies situated in Target stores [1–4]. Independent pharmacies (*n* = 4; 10.5%) included Banner Family Pharmacy, Fairmont Pharmacy, Phoenix Pharmacy, and S&G Drugs (Table 1).

**Table 1:** Pharmacy characteristics, Arizona pharmacy syringe purchase study, 2022 (*N* = 38)

Pharmacy type	*N* (%)
Chain	19 (50%)
Food Store	10 (26.3%)
Mass Merchandiser	5 (13.2%)
Independent	4 (10.5%)
*County*	
Maricopa	29 (76.3%)
Pima	9 (23.7%)
*Syringe sales outcomes*	
Always sold syringes (bags or box)	8 (21.1%)
Never sold syringes	17 (44.7%)
Limited sales to box only	3 (7.9%)
Sold at least once (bag or box)	21 (55.3%)
Sold at least twice (bag or box)	11 (28.9%)

There were 114 total purchase attempts (3 attempts at each of 38 pharmacies). When using discrete purchase attempts as the denominator, 24.6% (*n* = 28) resulted in the requested sale (bag of ten syringes) and 10.5% (*n* = 12) were limited to sales only of boxes of 100 syringes. When using pharmacies as the denominator, over half (55.3%, *n* = 21) of pharmacies sold syringes at least once, but only 8 pharmacies (21.1%) always sold syringes, whether in bag or box form.

Almost half (41.4%, *n* = 12) of Maricopa County pharmacies never sold syringes and over half (55.6%, *n* = 5) of Pima County pharmacies never sold syringes. Only one Pima County pharmacy always sold syringes (a mass merchandiser), but this pharmacy limited sales to boxes of 100 syringes. As Table 2 shows, in terms of pharmacy types, while only 26.3% (*n* = 5) of all chain pharmacies always sold syringes, 75% of Walgreens pharmacies specifically sold them every time. Only 1 CVS pharmacy always sold, with one of these sales limited to a box of 100 syringes. Among mass merchandiser pharmacies, none were found to never sell, and 2 of 3 Walmart pharmacies sold every time. While most independent pharmacies never sold syringes, one pharmacy (Independent 2) sold syringes every time.

**Table 2:** Arizona pharmacy syringe purchase study outcomes (*N* = 38), 2022

	Always sold	Sometimes sold	Never sold	Total
Chain	5 (26.3%)	6 (31.6%)	8 (42.1%)	19
CVS*	1	4	6	
Walgreens**	4	2	2	
Food Store	0 (0%)	4 (40.0%)	6 (60.0%)	10
Bashas Pharmacy	–	1	–	
Fry’s Pharmacy	–	1	5	
Safeway		2	1	
Mass Merchandizer	2 (40.0%)	3 (50.0%)	0 (0%)	5
Target/CVS	–	2	–	
Walmart Pharmacy	2	1	–	
Independent***	1 (25.0%)	0 (0%)	3 (75.0%)	4
Independent 1	–	–	1	
Independent 2	1	–	–	
Independent 3	–	–	1	
Independent 4	–	–	1	
Total	8 (21.0%)	13 (34.2%)	17 (44.7%)	38

*CVS is coded as chain and Target/CVS is coded as mass merchandizer due to setting differences

**Includes Community, a Walgreens Pharmacy

***Independent pharmacy names were suppressed to preserve confidentiality

### Pharmacy interaction

Buyers reported encountering two distinct types of pharmacy staff behaviors after requesting to purchase a bag of ten syringes, irrespective of sales outcome: negative staff interaction and positive staff interaction. Table 3 depicts interaction differences across an array of observed stages of engagement including (1) response to request, (2) customer query (question(s) asked of buyer), (3) normalization or othering of pharmacy service and customer, (4) response to purchase rationale, and (5) interaction closure.

**Table 3:** Pharmacy interactions following syringe purchase request, Arizona 2022

Negative Pharmacy Staff Interaction	Positive Pharmacy Staff Interaction
*Response to Request: Body Language and Tone of Voice*	
Shift to negative body language	Positive or neutral demeanor, No change
Initial verbal fumble, lack of clarity, pause	Confidence in exchange (request and response)
*Customer Query*	
Why are syringes needed? Do you have a prescription? Are you a patient here?	Why are syringes needed? What size do you need?
*Normalization or Othering of Pharmacy Service and Customer*	
Transfer to other staff Engagement (verbally or visually) with other staff	Same staff member handled interaction throughout
Syringe sale as not a pharmacy service: “We don’t do *THAT* here” Buyer is not a customer: “We save them for our customers”	Normalized interaction of care (If sale) behavior as with any pharmacy item sale
*Response to Purchase Rationale* *(“I need them to protect myself from HIV and Hepatitis C”)*	
Restatement of store policy Stating negative opinion about drug use, the area, or people who use drugs Dismissal-refer to another pharmacy	Expression of care Referral to other pharmacy Referral to syringe service program
*Interaction Closure*	
Pharmacy staff silence, staring at field investigator Investigator had to initiate closure: “thanks, goodbye” or “thanks, have a nice day”	Customary closure by staff: ‘thank you’ If no sale, “Sorry I could not help you”

While many pharmacy environments were initially reported as busy and, in a few cases “frantic,” purchase attempts almost always began with investigators being greeted as a customer by pharmacy staff with statements of: “*How can I help you*?”, “*Are you picking up*?” or “*Name and date of birth please*.” However, after the request to purchase syringes was stated, negative interactions included discernably changed vocal tone and body language and disconnection from eye contact with the field investigator.

*After I asked, ‘could I buy a bag of syringes?’ the body language completely shifted. The pharmacist began to then take steps backward as if I was going to rob her or jump over* (the) *counter.* (She) *immediately began to shake her head no. And I immediately felt hostile energy coming from her. The nonverbal communication was off the charts. Once the word syringes left my lips, it was a downhill experience from there.* (Case 006)

*Then I asked to buy a bag of syringes and she literally stopped and kind of almost jolted. Then she looked confused and flustered and then kind of stepped back and was like, “Oh, for a prescription? Do you have a prescription? What do you need them for?” It was just so awkward. And I said, ‘I need them to protect myself from Hepatitis C and HIV.” She said “Well, yeah, but it's our store policy….you can always try somewhere else…. I don’t know why people have been coming here lately.”* (Case 128)

*“I’d like to buy a bag of ten syringes.” And he squinted at me, slowed down, he paused for a really long time and he said, “Okay um,” and he did this ‘Um’ thing extremely long ….. his tone completely changed after I asked to buy the syringes to an aloof kind of uncaring kind of attitude towards me. …. When I said the HIV and HCV thing, he’s like, “well, I’m going to need a prescription* (from you)*.”* (case 043)

Conversely, during positive interactions, staff either maintained tone and body language or in a few notable cases, smiled, and adopted what field investigators described as “a more caring attitude.” In all cases, eye contact was maintained. Notably, when selling syringes, the staff member receiving the request for syringe purchase handled the entire exchange, which was experienced by investigators as normalized pharmacy interaction or “business as usual.” This interaction characterization was the case even when the outcome was not a syringe sale.

(He) *came around the corner and asked how he could help me. So instead of asking for my date of birth, he smiled and was polite and greeted me. I was like, “I would like to purchase a bag of ten syringes.” Immediately, he smiled and said, “Sure, no problem. We have 29 or 30 gauge, which would you prefer?” And I was like, “What?” And he is like, “Listen, we get a lot of people around here, and you look like you know what you want. And honestly, we want to keep everybody safe, so which would you prefer?” ……He was super kind and actually made me laugh at a few points.* (Case 093)

*Once I asked to buy the bag of syringes, the tone did not change. The nonverbal communication was 100% good. There was eye contact. There was sincerity in her voice. There was no negativity, no hostility at all. She just asked me what gauge, what size, what dosage I needed….and she said, “Okay, I’ll be right back.” She handled the entire interaction herself. She did not need to look to the pharmacist at all. She was on top of her stuff, so that made me feel good.* (Case 096)

In negative interactions, the exchange almost always involved a transfer to the pharmacist, encroachment by another staff member or a social exchange (verbal or nonverbal) between the staff person interacting with the investigator and other pharmacy staff overhearing the exchange. In all but one pharmacy field visit, there was no sale.

*I asked him if I could buy a bag of syringes. He paused and he said, “Is it for insulin?” His toned changed in the sense it wasn’t quite negative, but it was definitely questioning. He kind of shifted his weight to one side, and he looked at me, and then he looked back at the rest of the staff which weren’t paying attention to him at the time. And I said, “I need to protect myself against HIV and HCV.” And he just walked away to the back and talked to the pharmacist in the white coat, and then to another colleague, and other pharmacy techs….And I couldn’t hear what they were saying, but the woman standing behind the pharmacist kept looking over at me as he was talking.* (Case 103)

Following a request to purchase syringes, the next step in the exchange usually involved query of the buyer. In positive interaction pharmacies, the question was almost always “*What size* (or gauge)?” and in a few cases, these pharmacy staff would ask the purpose for purchase, and in response to the buyer, would follow immediately with an affirming “ok” and ask about the necessary gauge. In negative interaction pharmacies, the first question was usually “*Do you have a prescription*?” with similar iterations of “*Do you take insulin or testosterone*?” At that point, the buyer would state the reason for the syringe purchase.

In response to the buyer statement “*I need them to protect myself against HIV and Hepatitis C*,” a majority of staff in negative interaction pharmacies would state pharmacy policy prohibiting the sale without a prescription, and in two cases, these pharmacies reinforced the policy with a statement about the community around the pharmacy: *“We don’t sell them because of just this area, this location”* (Case 094). Notably, pharmacy staff in negative interaction pharmacies were generally unresponsive in tone and body language even after hearing the purchase rationale. In the interaction below (Case 104), the policy also prioritized specific health conditions.

**Table:** 

Buyer Statement	Pharmacy Staff Statement
*I would like to buy a bag of ten syringes*	*We are pretty much out of syringes right now and we only sell them by the box and you have to use them either for insulin or testosterone, and you need a prescription*
*You don’t actually legally need a prescription….to purchase syringes in Arizona*	*Mm-hmm* (affirmative), but because we run so low, we need to know what you need them for
*I need them to protect myself against HIV and HCV*	*We can’t sell them to you. We have to prioritize who we sell them to*

At this point in the communication exchange, the verbal and nonverbal staff communication sought to situate the exchange as either customary pharmacy practice or to characterize it as abnormal. In some cases, this behavior occurred immediately after the staff heard the initial syringe purchase request. In these cases, investigators reported staff lack of confidence about what to do next or how to respond, and in all cases, there was a staff transfer to the pharmacist or to what appeared to be a more senior pharmacy technician. These were generally nonverbal behaviors. The observation of one investigator was as follows:

*This is so simple, but it’s really, really important…if you’re rolling your eyes, you’re taking a step away from me, you’re turning your body this way to pretend to be on the computer, I’m already feeling like a piece of shit as a human. I don’t need you to do that, I need you to stay focused, even if it’s a – well, I don’t want it to be a ‘no’ – but I think that is huge for me, their body language*. (Case 008)

In negative interaction pharmacies, when denying syringe sale, staff said: ‘*We don’t do that here’* or *‘We don’t do that.’* Notably, this was followed by silence on the part of the staff member while maintaining gaze with the investigator. In these cases, this appeared to be the end of the interaction for the staff, but they did not walk away or close the discussion. For most of those cases, it was the investigator who felt they had to enact closure by saying ‘*thank you’* or even ‘*have a nice day*.’ This was even the case in some of the situations ending in syringe sale. For investigators, it appeared that by that time, they were clearly ‘*not a pharmacy customer*.’

*…*(and he said) *‘Yeah, no, we don’t do that without a prescription here.’ And then I just kind of stood there waiting to see if he would say anything else. And then it was awkward, just so awkward. That was it. And so I kind of waited. I thought maybe they* (pharmacist and technician) *might say something else. They did not. So I just said, “Okay, have a good day.” And I turned around and left.* (Case 130)

*….But then she just brought them* (syringes) *back, rang them up and didn’t say anything. She just handed me the bag….She did not say thank you. Actually, there was very little speaking….but I mean, she sold me the syringes, so that was nice.* (Case 134)

In contrast were staff behaviors characterizing the interaction as customary pharmacy practice. Nonverbal cues, verbal articulation, and methods of closure were distinctly different. As a result, investigators perceived a sense of humanity and care for their health, and they felt treated as pharmacy customers.

(At the end of the interaction) *she actually even said, “Please come back whenever you need some more.” Even though she was wearing a mask, I could tell she was smiling the entire time. It was great.* (Case 007)

(as the interaction was ending) *I actually thanked her for her customer service…. And she said “I don’t understand why folks need a reason to sell syringes, if you come in and buy them, you come in and buy them and that should be the end of it.” And just hearing what she had to say …. just let me know that she was about helping the community and she really cared about the work that she did.* (Case 096)

During positive purchase experiences, investigators reported that the pharmacist's tone and body language remained friendly and/or neutral, matching the pharmacist's actions by providing the syringes. For almost all investigators who shared this normalizing experience, no additional information was needed beyond the initial request to buy the syringes and the size/gauge. Such interactions were compared to buying chips, soda, or milk to illustrate how normal it felt.

*Yeah. It just seemed like I was just going in to purchase milk. I don't have to have a good reason to purchase milk. I want to purchase it and then I purchased it. So it was the same thing. I came in here to purchase syringes and I didn't have to have a good reason except I want them and I made the purchase. So that was great, it normalized syringe purchases.* (Case 088*)*

*I felt really refreshed walking out because I didn't even have to explain why I needed them. I didn't have to question him. It was kind of like no big deal, like I was buying a soda.* (Case 098)

### The emotional toll of syringe purchase attempts

As investigators continued to make syringe purchase attempts, it became undeniable that the experience demanded emotional labor and began to take a toll on them. The emotional impact was evidenced in their responses to questions regarding whether they were sold syringes or not, if they were treated like a customer, and how they felt when they left the pharmacy. Several investigators reported feeling unsettled by interactions with pharmacy staff in which the tone and behavior of staff did not match their actions.

*…this is a weird reaction emotionally* (for me) *because he was really friendly and remained neutral. It wasn't negative. And he smiled when he was telling me no. "Sorry, no." with a grin. But not in a ... I don't know. It was really odd and didn't feel right*. (Case 076)

Being refused by pharmacy staff the access to resources to protect themselves from harm while being presented with a seemingly a positive or friendly demeanor at points in the interaction was disorienting for investigators. This dissonance was noted and palpably felt by investigators, and it was their lived experience that appeared to be called forward in a self-stigmatizing manner.

*So I felt like crap… because they didn't sell me syringes. But their tone, their body language* (while positive)*, did not make me feel welcome, but it also didn't make me feel like I wasn't welcome. Oh my God, that's so confusing if...I don't know. So their tone, and their body language, was, they were flustered, and fumbling over words. Yes. But they didn't make me feel like I was scum. But I also didn't feel welcome there. That's what I'm trying to say.* (Case 089)

Some investigators noted the incongruity that a healthcare provider (pharmacy) had the power to determine their access to a legally available tool to prevent bloodborne illness; and that their status as a PWID was the reason they were denied this tool. Because investigators were trained to directly state the rationale of protecting themselves from HIV and Hepatitis C, both staff and investigators were forced to confront the reality of what refusal meant for the health of the person denied access to sterile syringes.

*How can they be okay with sending someone off to get disease? At least the other place said, "Well, what about Walgreens?" Like, "Why don't you go to Walgreens?" But it's also like, why am I excited about a crumb? Like, "We can't help you, but here's a crumb.” …But I feel like it breaks you down, you know? And you're just groveling and anything they give you is like, "Oh wow, they treated me so good," when they actually didn't treat me well and they didn't treat me like a pharmacy customer. How'd I feel when leaving the pharmacy? Defeated, enraged, annoyed, upset, disgusted, powerless, fearful that like this is where we're at.* (Case 086)

The cognitive dissonance became morbid when one pharmacy suggested that the investigator return once they have Hepatitis C or HIV infection, such that HIV or HCV prevention was not going to be a pharmacy service but treatment once infected would be. The case below describes a buyer encounter with a pharmacist who refused to sell sterile syringes, yet took the time to explain which medications were available if and when the investigator were infected with HIV and/or hepatitis C.

*Her tone was very pleasant and friendly… She said they cannot sell them unless I have a prescription….She did, however, say, "You mentioned Hep C, have you been tested for it or anything?" And I said no. And she educated me on the medications* (the pharmacy) *offered and things like that.*… *And she said, "A lot of insurance cover it now.”* (Case 047)

In this field case, the investigator felt as if they were treated like a customer because of the helpful education by the pharmacist. Several investigators made statements about the purchase attempt experience suggesting that expectations are incredibly low among people with lived experience who are seeking to protect themselves from harm with the help of pharmacies. The impact of repeatedly being separated from “normal” pharmacy customers, and obvious expressions of stigma appeared to become internalized, and investigators themselves began to question if they were worthy of safety or bodily protection. Other responses echoed this sentiment of “being thankful for crumbs” or expressing gratitude despite being refused a sale. Being denied access to sterile syringes signaled a lack of worthiness as a human being, while getting a sale signaled worthiness.

*Well so far, my experience with the pharmacies in these past few days have been unpleasant and dehumanizing, so I am like, hmm, this just seems to be the landscape of trying to purchase syringes in Arizona. I was like, they just don't care about folks that use drugs. …..The pharmacist said, "We can't, we can't sell them to you. We have to prioritize who we sell them to." And I said, "Okay, well thank you." So that was that. Which also made me feel really dehumanized. Like you're going to prioritize other folks' needs above my own? And how do they even come up with that decision? Why is one life worth more than the other? I don't know, it was really upsetting, honestly.* (Case 104)

Some buyers were left wondering why they felt so pleased with the outcome of an experience that was normalized, signaling awareness of internalized stigma.

*So it was a good experience. When I was leaving the pharmacy, I felt great too. I was proud of myself, a little ashamed that I felt this way in the beginning…You’re out here trying to combat the stigma, and then you’re feeding right into it. Come on! So I was a little ashamed of myself for those feelings or those thoughts. Overall, I was happy that I overcame it, and I made the purchase. So I felt good leaving.* (Case 097)

## Discussion

To our knowledge, this Arizona-based syringe study is the first to characterize pharmacy interactions and the subjective experience of field investigators who have lived/living drug use experience. This is important because the stated purchase rationale of protecting oneself from HIV and hepatitis C was embodied by the lived experience of the field investigators—without which the measured subjective experience would not have been appropriately captured. Centering such experiences reflects the ethics of secret shopper studies generally and is likely why they have been cited as such potent methods to study designs that assess potential barriers to health equity [32]. Audit studies whereby people call pharmacies asking for the availability of certain medications have also been conducted [33]. This study where the real-life interaction is experienced and characterized provides additional and critical information for future pharmacy training to improve harm reduction pharmacy practice.

Findings reinforce the documented trend of reduced pharmacy syringe sales over time, as only 24.6% of purchase attempts in this study resulted in a sale of sterile syringes without limitations. This finding is notable because pharmacy accredited education, public health messaging, evidence-based pharmacy interventions, and state policy encourage harm reduction pharmacy practice including the sale of syringes. Unfortunately, in many cases, it appears that pharmacies and/or pharmacy staff members may decide to deny selected services to a portion of their clientele based on either stigma or their disapproval of its intended use. And these beliefs driving decisions to deny syringe sale appears to trump any disapproval of their role in structurally facilitating bodily harm to people by gatekeeping resources. Further, it remains unknown whether pharmacies even considered investigators their clientele. The othering of the person who requests harm reduction services such as syringes has also been found with decisions by pharmacists to limit the dispensing of Buprenorphine for opioid use disorder if the people presenting the prescriptions are new clients or ‘not local’ [34]. All field investigators in this study were initially greeted as any customer would be, and it was only after the request to purchase syringes that the investigators were treated differently. This indicates that the othering resulting from these negative interactions could likely have derived from stigma towards people who use illicit drugs and prejudice enacted by pharmacy staff.

Pharmacists demonstrated discretion to act around issues regarding safety, as well as legal and ethical obligations to act in patients’ best interests. Pharmacists and pharmacy staff members operate in a complex landscape reflecting interactions of laws, professional norms, practice standards, and ethics. However, the reasoning that informs the exercise of discretion by pharmacists and pharmacy staff is important. A recent viewpoint published in *JAMA Internal Medicine* suggested that misunderstandings or misperceptions of syringe access among pharmacists may contribute to instances where pharmacists elect to refuse syringe sales [35]. Pharmacists may not be aware of the study by Davidson et al. demonstrating that increased pharmacy sales of sterile syringes were not associated with deaths in the nearby communities [36]. Pharmacists have also been urged to use their role to provide consistent and immediate access to sterile syringes by professional organizations such as the American Pharmacists Association (APhA), which has issued clear policy statements in support of harm reduction strategies [37]. So this appears to be an issue at pharmacist, pharmacy or pharmacy chain levels.

Syringe sales are associated with the prevention of infections among PWID in the US, and it is concerning that pharmacy discretion resulting in refusal may contribute to the increased prevalence thereof. Thus, state public health policy goals to reduce or eliminate hepatitis C may be confounded by the reluctance, or as this study observed, outright refusal by many pharmacies to engage in preventative healthcare strategies. In some cases, the solution may indeed be continuing education focused squarely on negative attitudes by healthcare service providers writ large about injection drug use due to the deleterious health outcomes that have been shown to associate with future health-seeking behavior once faced with such stigma. Negative and stigmatizing experiences took an emotional toll even on trained investigators who in some cases were years away from their lived experience. Specifically, the reported jarring effect of being told they are not deserving of bodily safety and protection against HIV and HCV, but (in one case) deserving of treatment after infection, reflected the level of experience reported in the 2019 Arizona study [25]. The potentially deadly outcome in that case was participant decision to skip future mistreatment in the pharmacy and reuse syringes or share them [25]. This reflects other studies where the anticipation of abusive and stigmatizing behavior from healthcare providers results in strategies to avoid such encounters [6, 7, 38]. Pharmacists are not uniform in their beliefs regarding harm reduction, though steps toward establishing harm reduction latent class assignments may help education and training interventions target pharmacists and staff based on their group placement [26, 39].

Education, however, is likely not sufficient to appreciably change pharmacy practice. Policymakers at the state level (as states govern pharmacy policy in the US) should address the question of whether pharmacies are important harm reduction health partners for the prevention of bloodborne illnesses in Arizona and elsewhere. If this is deemed the case, lawmakers, pharmacy boards and pharmacy licensing authorities will need to identify evidence-based structural incentives to assure pharmacy behavior and capacity to accomplish harm reduction services. A prime example of harm reduction policy is the state standing order for naloxone dispensing. One Indiana study found that the availability and dispensing of naloxone increased in the 2 years following the implementation of a statewide standing order [40, 41]. Because the lack of a prescription was often cited as a policy requirement or used as a screen for syringe sales denial by pharmacy staff, implementing a standing order for syringes for the prevention of bloodborne illnesses could be another potentially helpful structural intervention.

The relevance of education and structural interventions extends beyond sterile syringe sales. Studies have shown that Buprenorphine for opioid replacement therapy, birth control, emergency birth control, and access to hormones for transgender patients, have all been targets of pharmacist gatekeeping in ways that are clearly not evidence-based nor aligned with patients’ medical and healthcare best interests [39, 41]. Educational interventions should address misperceptions related to a need for more information, personally held beliefs, prejudice, or other factors not considered here.

## Limitations

It is essential to acknowledge limitations that shape the scope of our findings. First, the relatively modest sample size restricts our ability to confidently compare syringe availability among different pharmacy types within the state. However, all pharmacies were visited in the selected areas, and this suggests that study findings are relevant for those pharmacies. Furthermore, the study's geographical focus on Arizona limits the generalizability of our results to other US states, especially for our novel findings regarding the pharmacy staff interaction with investigators. Sales outcomes unfortunately reflect the insufficient availability of sterile syringes through pharmacies given the alignment with findings from other studies. Additionally, we recognize that purchaser-specific factors, such as demographic characteristics, appearance, and demeanor, could have influenced the outcomes. This last noted intersectional bias, however if true, is yet another reason to address these issues structurally.

## Conclusions

Pharmacies miss or purposely avoid opportunities to advance efforts to prevent and eliminate bloodborne infections by refusing to sell syringes to individuals without a corresponding prescription. State regulatory policy facilitating pharmacy syringe sales and targeted pharmacy-staff level education may help advance the achievement of public health goals to eliminate bloodborne infections in Arizona.

### Supplementary Information

**Additional file 1.** Buy Study Field Researcher Instrument.

## Data Availability

The field researcher instrument has been included with the manuscript as Additional file 1 for publication.
