# Supplementary material for: Come back when you’re infected: pharmacy access to sterile syringes in an Arizona Secret Shopper Study, 2023
Source: Harm Reduct J. 2024 Feb 22;21:49. doi: 10.1186/s12954-024-00943-w (PMC10885601; doi:10.1186/s12954-024-00943-w)
Supplement: Supplementary file 1 — Additional file 1. Buy Study Field Researcher Instrument. [file 12954_2024_943_MOESM1_ESM.pdf]

Buy Study Field Researcher Instrument

When using or adapting items from this instrument, please use the following citation:
Russell DM, Meyerson BE, Mahoney AN, Garnett I, Ferrell C, Newgass K, Agley JD, Crosby RA, Bentele KG, Vadiei N, Frank D, Linde-Krieger LB. Come Back When You're Infected: Secret shopper study of pharmacy syringe sales in Arizona, USA 2023. *BMC: Harm Reduction Journal* 2024.

Please record your responses to these questions using the audio recorder within 30 minutes of your pharmacy visit and before you visit another pharmacy.

1. Please share your name, the date and the time you visited the pharmacy.
2. Please share the name and street address of the pharmacy you just visited.
3. Before you entered the pharmacy – did you think you would be treated well there? (without any stigma?)
    a. Please explain why you expected or did not expect to be treated well.
4. Please describe the pharmacy environment when you walked into the pharmacy. Be as descriptive as possible and please share what you felt during your pharmacy entry. Please also share how you felt entering that space.
5. How many customers were at the pharmacy? (If this is a larger store where a pharmacy exists, please share only the number of people at the pharmacy only).
6. What was it like for you to be at the pharmacy counter? Be as descriptive as possible.
    a. What were you thinking and feeling while you were waiting to be served by the pharmacy staff?
7. How many pharmacy staff people were working at the pharmacy when you were there? (estimate if needed).
8. Who initially greeted you at the pharmacy counter? (Pharmacy technician? Pharmacist?)
    a. Pharmacists usually wear a white medical coat. Pharmacy technicians are usually in scrubs or some other street clothing.
9. What was their 'tone' with you initially (before you asked to buy syringes)?
    a. Please describe.
    b. Please rate their tone:
        i. hostile (openly aggressive)
        ii. negative (sense judgement and disgust even if nothing is directly),
        iii. neutral
        iv. friendly

10. What happened once you asked to buy a bag of 10 syringes? Be as descriptive as possible.

11. What was their 'tone' *after* you asked to buy the syringes?

    a. Please describe.

    b. Please rate their tone:
        i. hostile (openly aggressive)
        ii. negative (sense judgement and disgust even if nothing is directly),
        iii. neutral
        iv. friendly

    c. Please share other non-verbal communication that you recall.

12. Did the staff person handle the entire interaction themselves or did they pass it to another staff person (maybe even a pharmacist)?

    a. Please answer Yes or no and also describe what happened.

13. How did you feel during this part of the interaction?

14. Did the pharmacy sell you a bag of 10 syringes? If not, did they sell you any syringes (less than 10)?

If they sold you syringes (answer questions 15, 16 and 17): (if not skip to question 18)

15. Did the pharmacy require any form of identification for the purchase? (Yes or no)

    a. Please describe what they wanted

16. Did the pharmacy require you to sign a document or form of some kind to purchase? (yes or no)

    a. If yes, please describe what they required.

17. Did the pharmacy staff discuss safe syringe disposal or safe syringe use? (yes or no)

    a. If yes, please describe what they told you.

Now Skip to question 19.

If they did not sell you syringes (answer question 18)

18. What was the reason given for not selling you syringes?

19. How did the interaction with the pharmacy staff end? (after selling or not selling the syringes?) Please describe.

20. In your opinion, did the pharmacy staff treat you like a pharmacy customer?

21. Please describe as much as you can about how the pharmacy staff treated you from the time they greeted you through the entire interaction.

22. How did you feel when you were leaving the pharmacy?

23. Approximately how long was the interaction from request to buy to the time you left the pharmacy counter?

    a. 2 minutes or less
    b. Between 3-5 minutes
    c. Between 5-10 minutes
    d. More than 10 minutes

24. If you ever needed to purchase syringes in the future, would you go to this pharmacy to get them?

25. Please share anything else important to you about this experience.

Please upload your audio recording to the website [list URL] within 24 hours

Thank you so much!
